# Context matters: the evolving use of biomarkers in Alzheimer's disease care

**DOI:** 10.1016/j.ebiom.2024.105387

**Published:** 2024-10-03

**Authors:** Juan Fortea

**Affiliations:** a Sant Pau Memory Unit, Institut de Recerca Sant Pau, Universitat Autonoma de Barcelona, Barcelona, Spain; b Network Center for Biomedical Research in Neurodegenerative Diseases (CIBERNED), Madrid, Spain; c Catalan Down Syndrome Foundation, Barcelona, Spain

Biomarkers play a crucial role in advancing our understanding of Alzheimer's disease (AD). These measurable indicators enable the detection of the presence and progression of the disease at earlier stages, therefore improving patient outcomes and providing opportunities for timely intervention and more personalized treatment plans.^1^ This series aims to provide a forward-thinking perspective on the current state of biomarker research and the challenges we face in implementing these tools in clinical practice and clinical trials. We will examine the critical importance of the context of use, how different clinical settings and patient populations impact the utility of biomarkers.

Biomarker research in AD has traditionally focused on sensitivity and specificity, particularly through the AT(N) classification framework^2^ that categorizes biomarkers into ‘positive’ or ‘negative’ groups for diagnostic purposes. However, in clinical practice, the utility of these biomarkers will critically depend on their positive and negative predictive values, which are significantly influenced by the prevalence of the condition within specific populations. This means that a biomarker's performance can vary widely across different healthcare environments, such as the general population, primary care, or specialized centers, highlighting the need for context-specific evaluations.^3^ Furthermore, transitioning biomarkers from research settings to routine clinical care require addressing issues such as standardization, pre-analytical and analytical conditions. The work of Schöll et al.^4^ discusses these challenges together with adapting these biomarkers for different healthcare environments, ranging from specialized memory clinics to primary care and (future) community screening programs.

Our series highlights two specific clinical scenarios that further underscore the importance of considering the context of use. First, Carmona-Iragui et al.^5^ examine the use of biomarkers in genetically determined forms of AD, such as autosomal dominant AD and Down syndrome. In these cases, all individuals are considered to have AD by definition.^2^ As a result, biomarkers do not serve to differentiate between disease presence or absence but are instead utilized to monitor disease progression. In these contexts, biomarkers with wide dynamic ranges, like plasma GFAP or p-tau, offer opportunities to serve more than one purpose. They may require distinct thresholds depending on their intended use, whether for identifying amyloid-positive individuals who are asymptomatic or for distinguishing between asymptomatic and symptomatic stages of the disease.^6^ Second, McFeely et al.^7^ explore the use of biomarkers in individuals with intellectual disabilities (ID), many of whom are at an increased risk for neurodegenerative diseases. Diagnosing AD in this population is particularly challenging, as symptoms may be masked by the underlying intellectual disability. Reliable biomarkers could significantly improve diagnostic accuracy and access to disease-modifying therapies for these individuals. However, current diagnostic frameworks often exclude people with ID, and the interpretation of biomarkers may be complicated by conditions associated with different forms of ID that can affect biomarker levels.

In another understudied yet crucial area, McGlinchey et al.^8^ examine the use of biomarkers in the Global South, where diverse genetic, cultural, and socioeconomic contexts present both challenges and opportunities. While regions like Latin America, Africa, and South-East Asia face obstacles such as limited infrastructure, funding, and harmonization, they also offer unique opportunities for advancing global dementia research. Understanding these regional factors is essential to ensure equitable access to diagnostic tools and therapies worldwide.

Beyond their role in diagnosis, biomarkers can be applied to a range of purposes, with clinical trials being one of the most critical. Pascoal et al.^9^ cover the expanding role of biomarkers in drug development. Incorporating biomarkers into clinical trials is increasingly crucial, serving multiple purposes such as identifying suitable participants, demonstrating target engagement, monitoring disease progression and side effects, and assessing therapeutic efficacy. Biomarkers are essential tools that can significantly accelerate the development of effective treatments for AD and related dementias. Looking ahead, if certain biomarkers are validated as surrogates for clinical efficacy, their importance will only grow, particularly in the context of preclinical AD, where demonstrating clinical effects is inherently challenging.

Finally, the series explores the ethical considerations of using biomarkers, as discussed by Karlawish et al.^10^ Even when access to the latest treatments is limited or unavailable, patients and families may still benefit from knowing the cause of cognitive impairments and understanding their prognosis. This raises the question of whether biomarker-derived diagnostic information should be shared with patients to support informed decision-making and future planning, despite the lack of treatment options. The debate underscores the tension between the value of providing diagnostic knowledge and the potential psychological impact of knowing one's prognosis without available therapies.

Collectively, the articles in this series highlight the transformative potential of biomarkers in advancing the diagnosis and treatment of AD, while also revealing the complexities of integrating these tools into clinical practice. This series also demonstrates that while biomarkers are set to become indispensable in managing AD, they cannot replace the need for clinical judgment in the different contexts of use.

## Declaration of interests

J.F. reported receiving personal fees for service on the advisory boards, adjudication committees or speaker honoraria from AC Immune, Adamed, Alzheon, Biogen, Eisai, Esteve, Fujirebio, Ionis, Laboratorios Carnot, Life Molecular Imaging, Lilly, Lundbeck, Perha, Roche and outside the submitted work. O.B., D.A., A.L. and J.F. report holding a patent for markers of synaptopathy in neurodegenerative disease (licensed to Adx, EPI8382175.0).
